# Exploring Neural Stem Cell Therapies as Innovative Treatments for Glioblastoma

**DOI:** 10.1007/s10571-025-01619-0

**Published:** 2025-11-20

**Authors:** Marola Fawzy, Hend M. ElTayebi, Amany Samir

**Affiliations:** 1 School of Life and Medical Sciences, University of Hertfordshire Hosted By Global Academic Foundation, New Administrative Capital, Egypt; 2 Department of Pharmacology and Toxicology, Faculty of Pharmacy and Biotechnology, German University in Cairo, Cairo, Egypt; 3 School of Life and Medical Sciences, University of Hertfordshire Hosted by Global Academic Foundation, New Administrative Capital, Egypt

**Keywords:** Neural stem-cells, Glioblastoma treatment strategies, Brain tumor, Personalized therapy

## Abstract

Glioblastoma (GB) is among the most aggressive and treatment-resistant brain tumors, largely due to its heterogeneous tumor microenvironment (TME) and the protective nature of the blood–brain barrier (BBB). Recent advances have highlighted the therapeutic potential of neural stem cells (NSCs), which possess tumor-homing capabilities that enable them to selectively migrate toward and infiltrate GB sites. Engineered NSCs can deliver therapeutic agents, including oncolytic viruses, prodrug-converting enzymes, and genetic materials, offering targeted treatment while minimizing systemic toxicity. Preclinical studies have demonstrated NSCs' promise in enhancing drug delivery, modulating the TME, and promoting anti-tumor immune responses. However, translational hurdles persist, including tumor heterogeneity, species-specific immune responses, and challenges in ensuring long-term safety. Emerging strategies—such as genetic modification to improve tumor targeting and the incorporation of biomaterials to enhance retention—are under investigation. Integrating personalized medicine approaches may further optimize NSC-based therapies by tailoring treatment to individual patient profiles. While significant barriers remain, ongoing research may ultimately establish NSCs as a viable and effective platform for GB therapy.

**Graphical Abstract:**

*Engineered NSC for GB* GB, an aggressive brain tumor, is being tackled with neural stem cells (NSCs) that are engineered to infiltrate tumor sites, showing potential to enhance drug delivery and modulate the tumor microenvironment for future personalized treatments. Despite the challenges, ongoing translational research actively addresses tumor heterogeneity. 
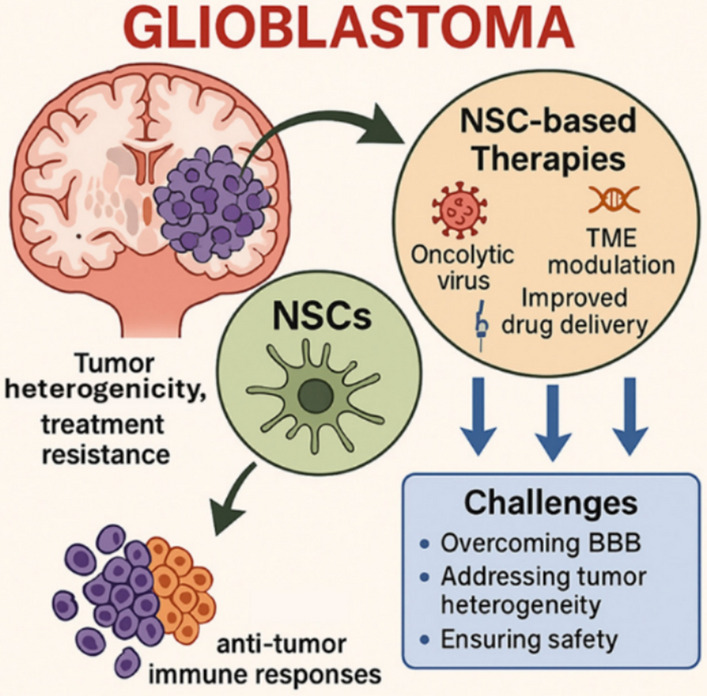

## Introduction

Cancer encompasses a diverse range of diseases characterized by the uncontrolled growth of abnormal cells, which can invade surrounding tissues and metastasize to distant organs (WHO 2025). It remains the second leading cause of death worldwide, with its burden projected to rise over the next two decades, driven by population aging, unhealthy lifestyles, and environmental changes, particularly affecting low- and middle-income countries (Qiu et al. 2021; Wu et al. 2024). In 2022, approximately 20 million new cases and 9.7 million deaths were reported globally, including 150,578 new cases and 95,275 deaths in Egypt, where brain cancers accounted for 4,452 cases and 3,611 deaths (Bray Bsc et al. 2024; Ferlay et al. 2024). Among primary intracranial tumors, gliomas are the most common in adults, arising from glial cells that support neuronal function, with glioblastoma (GB) representing the most aggressive form. They exhibit considerable heterogeneity in histopathology, molecular features, clinical behavior, and prognosis. Fortunately, classification of gliomas has evolved from early twentieth-century histological characterization to the 2021 WHO CNS5 system, which integrates molecular markers such as IDH mutation status, EGFR amplification, TERT promoter mutations, and combined chromosome 7 gain/chromosome 10 loss (+ 7/– 10) with histological criteria for improved diagnostic and prognostic accuracy (Liguori 2024; Pinheiro et al. 2023; Rios et al. 2024; Torp et al. 2022).

Among gliomas, GB represents the most aggressive and prevalent subtype, accounting for approximately 57% of all gliomas and nearly half of all malignant primary brain tumors. GB is characterized by rapid progression, resistance to therapy, and poor prognosis, with a median survival of only 12–15 months despite aggressive multimodal treatment. Moreover, it is more common in older adults, particularly those aged between 74 and 85, and shows a higher incidence in males. Clinically, GB presents with nonspecific symptoms such as persistent headaches, nausea, vomiting, focal neurological deficits, cognitive impairment, and seizures, mainly due to increased intracranial pressure and tumor infiltration. Histopathologically, GB is classified as a grade IV astrocytoma and exhibits hallmark features including hypercellularity, nuclear atypia, high mitotic activity, microvascular proliferation, and necrosis. In addition to its histological complexity, GB pathogenesis involves extensive molecular heterogeneity, including alterations in key oncogenic pathways such as receptor tyrosine kinase (RTK), p53, and retinoblastoma (RB), as well as an immunosuppressive and pro-angiogenic tumor microenvironment (TME) driven by hypoxia-induced vascular endothelial growth factor (VEGF) expression and the recruitment of tumor-associated macrophages (TAMs). Moreover, GB cells exhibit the presence of a stem-cell–like phenotype and, in IDH-mutant tumors, relapse is characterized by a marked expansion of proliferating stem cells. This expansion is associated with acquired alterations in cell cycle regulatory genes, including CDKN2A deletions and CCND2 amplifications. These features not only facilitate tumor growth and invasion but also contribute to therapeutic resistance, underscoring the need for novel and more effective treatment strategies (Ma et al. 2021; McKinnon et al. 2021; Pinheiro et al. 2023; Rios et al. 2024; Sipos et al. 2025; Tan et al. 2020; Varn et al. 2022).

The current therapeutic management of GB is multifaceted, typically necessitating a multimodal approach tailored to individual patient characteristics. Despite ongoing research and the exploration of novel treatment modalities, existing strategies remain the subject of debate due to variable efficacy. Furthermore, the overall prognosis continues to be poor, largely attributed to the tumor’s high rate of recurrence and resistance to conventional therapies (Lowe et al. 2019).

Surgical resection is a cornerstone of GB management, aiming for maximal safe tumor removal to alleviate symptoms and improve overall (OS) and progression-free survival (PFS). While greater resection extent correlates with better outcomes, complete excision is hindered by GB’s infiltrative growth and proximity to critical cortical areas. Advances such as intraoperative MRI and 5-aminolevulinic acid (5-ALA) fluorescence have improved precision, yet surgery alone is insufficient, necessitating adjuvant radiotherapy and/or chemotherapy to address residual disease and reduce recurrence (Bonosi et al. 2023; Gerritsen et al. 2022; Patel and Chavda 2024).

Post-surgical management of gliomas typically combines radiotherapy and chemotherapy. Nonetheless, complete excision is often unattainable due to tumor aggressiveness and proximity to critical brain areas (Chiariello et al. 2023). Radiotherapy, on the other hand, targets residual malignant cells via DNA damage from high-energy beams, reaching deeply invasive cells. However, its efficacy is limited by glioma cell resistance and the hypoxic tumor microenvironment (TME), and it carries significant side effects, such as alopecia, infertility, leukoencephalopathy, and neural tissue damage, that impair patients’ quality of life (Liu et al. 2023).

Similarly, chemotherapy for glioma faces major hurdles, notably BBB penetration and efflux mechanisms that limit drug accumulation in the brain (Luiz et al. 2021). Temozolomide (TMZ), the FDA-approved first-line agent, crosses the BBB but has a short half-life (~ 1.8 h), requiring high systemic doses. In addition to hydrolyzing under physiological conditions to form a methyldiazonic cation, inducing cytotoxicity via G2/M-phase arrest. Nonetheless, recurrence is frequent due to resistance mechanisms in GB cells (Ortiz et al. 2021; Sayiner et al. 2020). In addition, bevacizumab, an FDA-approved monoclonal antibody against VEGF, inhibits angiogenesis and thereby restricts tumor growth (Gerriets and Kasi 2023). Lomustine and carmustine are also approved for glioma but have declined in use due to high toxicity and limited benefits over radiotherapy (Fisher and Adamson 2021). Paclitaxel (PTX), effective in other cancers via microtubule stabilization–induced apoptosis, has limited glioma use due to poor BBB penetration, high lipophilicity, and significant cytotoxicity (AbdEl-haq et al. 2023).

Furthermore, tumor treating fields (TTFields) are a non-invasive GB therapy delivering low-intensity, intermediate-frequency alternating electric fields via scalp-mounted arrays to disrupt mitotic spindle formation, inhibit tumor growth, and induce apoptosis in dividing cells while sparing healthy tissue. Clinical trials have confirmed their efficacy and safety in newly diagnosed (ndGBMs) and recurrent GBs (rGBMs), establishing TTFields as the fourth major modality alongside surgery, radiotherapy, and chemotherapy (Voloshin et al. 2020). However, adoption remains low (< 12% for ndGBMs, < 16% for rGBMs) due to high cost, the need for ≥ 18 h daily wear, continuous scalp shaving, and associated impacts on adherence, self-esteem, and quality of life. Despite promising results, their mechanisms, synergy with standard treatments, and predictors of response remain under investigation (Guo et al. 2022; Pinheiro et al. 2023).

**Fig. 1:**
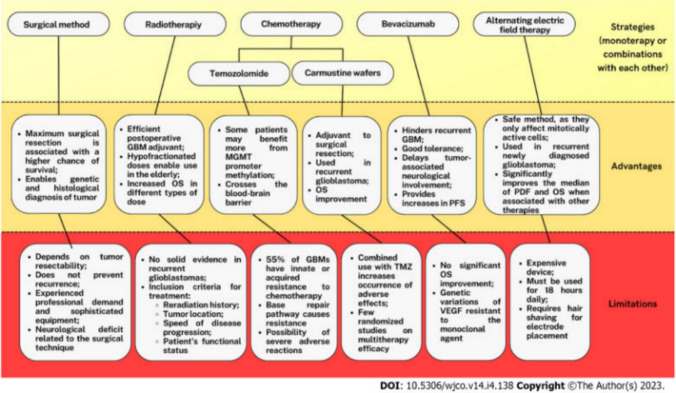
Overview of current GB therapies, outlining benefits, limitations, and key terms (Pinheiro et al. 2023)

Due to the significant and often intolerable side effects and limitations associated with current treatment modalities, as illustrated in Fig. 1, immunotherapy has emerged as a promising approach in the treatment of GB, aiming to overcome the limitations of conventional therapies by harnessing the body’s immune system to target and eliminate tumor cells. Despite the highly immunosuppressive microenvironment of GB, several immunotherapeutic strategies have demonstrated potential in preclinical and early clinical studies. These include immune checkpoint inhibitors (ICIs), chimeric antigen receptor (CAR) T cell therapies, dendritic cell (DC)-based vaccines, and oncolytic viruses (OVs). Each approach seeks to modulate immune responses to enhance tumor recognition and destruction while minimizing off-target effects (Pinheiro et al. 2023; Segura-Collar et al. 2023). However, the clinical success of immunotherapy in GB remains limited due to challenges, such as tumor heterogeneity, limited immune cell infiltration, and the blood–brain barrier. Hence, ongoing research is focused on identifying predictive biomarkers, optimizing delivery methods, and developing combination therapies to improve patient outcomes (Dunn et al. 2020).

While current treatment options for brain tumors remain limited, advances in molecular biology have significantly contributed to the development of targeted therapies and immunotherapeutic strategies resulting in improved prognoses (Li et al. 2023). Despite these advancements, the therapeutic efficacy of such approaches is frequently undermined by low response rates and the eventual development of drug resistance. These limitations continue to present major obstacles to the advancement of effective treatment modalities for brain tumors (Liguori 2024).

**Fig. 2:**
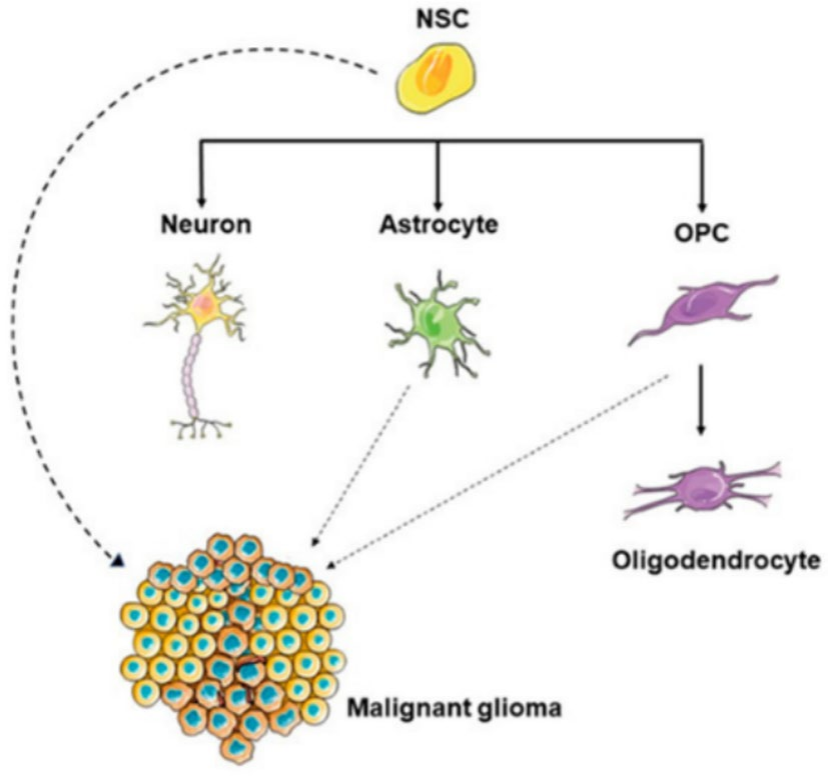
Neural stem cells (NSCs) are undifferentiated, multipotent cells capable of both self-renewal and differentiation into various neural lineages, including neurons, astrocytes, and oligodendrocyte precursor cells (OPCs). Under certain pathological conditions, genetic or epigenetic alterations in NSCs can drive their transformation into glioma stem cells (GSCs), which are believed to play a central role in the initiation and propagation of gliomas. (Loras et al. 2023)

Henceforth, over the past 15 years, neural stem cells (NSCs) have garnered substantial scientific and commercial interest due to their remarkable biological properties. Their inherent plasticity, combined with their capacity for unlimited self-renewal, positions them as a promising therapeutic tool (Ottoboni et al. 2020). NSCs are present within the ventricular system during CNS development, they become regionally restricted in the adult brain to two neurogenic niches: the subventricular zone (SVZ) and the Sub-granular zone (SGZ) of the hippocampal dentate gyrus (Navarro Negredo et al. 2020; G. L. Zhang et al. 2021). These NSCs possess the capacity to differentiate into glial lineages—such as astrocytes and oligodendrocytes—as well as neurons (Ottoboni et al. 2020). While the majority of progenitor cells undergo terminal differentiation by the end of embryogenesis, oligodendrocyte precursor cells (OPCs) remain undifferentiated and retain proliferative capacity throughout adulthood. Moreover, differentiated astrocytes exhibit limited proliferative potential, particularly in response to injury-induced stimuli. Due to their regenerative properties and proliferative capabilities, NSCs, OPCs, and reactive astrocytes are regarded as potential cellular sources for gliomagenesis (Fig. 2) (Kiaie et al. 2022; Loras et al. 2023).

**Fig. 3:**
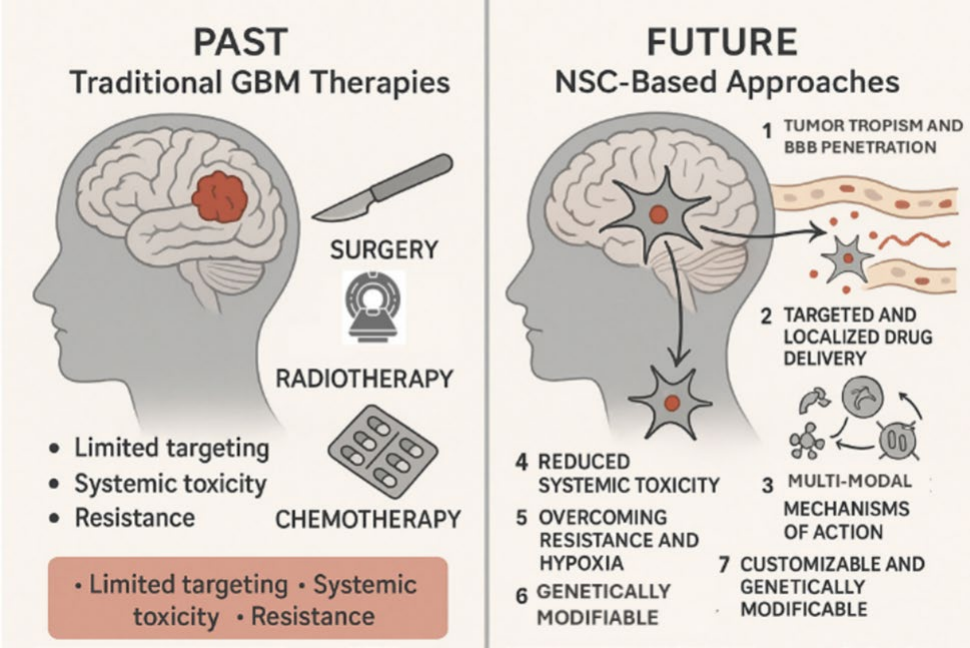
Limitations of conventional GB treatments and the potential advantages offered by neural stem cell (NSC)-based therapies

Therefore, this review anticipates the critical analysis of emerging therapeutic strategies involving the application of neural stem cells in the treatment of GB. By exploring recent advancements in this field, this review aims to highlight the potential of neural stem cell-based therapies to overcome current limitations in GB management and contribute to the development of more effective and targeted treatment modalities *(*Fig. 3*)*.

## Neural Stem Cells in Glioma genesis

The origin of gliomas remains a central question in neuro-oncology, with accumulating evidence pointing toward NSCs as a potential initiating cell population. NSCs, residing primarily in the SVZ and SGZ, possess life-long self-renewal capacity and multipotency, enabling them to generate neurons and glia throughout adulthood. These same characteristics, however, also make NSCs vulnerable to oncogenic transformation under conditions of genetic instability or dysregulated signaling (Hamed et al. 2025).

Core developmental signaling pathways, including Notch, Wnt/β-catenin, and Sonic Hedgehog (SHH), play indispensable roles in NSC maintenance and lineage specification. In gliomas, dysregulation of these pathways is a frequent molecular hallmark. Notch hyperactivation preserves stem-like phenotypes and impedes differentiation; aberrant Wnt signaling facilitates unchecked cellular proliferation; and constitutive SHH activity drives NSC expansion, thereby augmenting tumorigenic capacity (Genet et al. 2023; Torrisi et al. 2021). Concurrently, mutations in critical tumor suppressor genes (TP53, PTEN, NF1) and oncogenes (EGFR, IDH1/2) disrupt NSC regulatory networks, predisposing these cells to malignant transformation (Pouyan et al. 2025). This causal relationship is supported by genetically engineered mouse models (GEMMs), wherein the introduction of such mutations into NSCs residing in the SVZ consistently yields high-grade gliomas, thereby providing direct experimental evidence for the involvement of NSCs in gliomagenesis (Noorani 2019).

An alternative hypothesis posits that gliomas may arise not exclusively from NSCs, but from more differentiated glial populations such as astrocytes or OPCs that undergo oncogenic reprogramming to reacquire stem-like properties. Among these, OPCs are of particular interest, as they retain inherent proliferative potential and can give rise to tumors when harboring glioma-associated mutations (Zhuang et al. 2022). Comparative lineage-tracing studies have revealed that introducing identical mutations into NSCs versus OPCs produces gliomas with distinct transcriptomic profiles and histopathological characteristics, indicating that intertumoral heterogeneity may, in part, be attributable to differences in the cell of origin (Ah-Pine et al. 2023; Faisal et al. 2025).

Overall, although accumulating evidence substantiates a central role for NSCs in gliomagenesis, parallel oncogenic origins from more differentiated glial populations remain equally plausible. The diversity of potential initiating cells likely underlies much of the biological and clinical heterogeneity observed in gliomas, underscoring the necessity of refining origin-based classification frameworks to inform precision therapeutic strategies.

## Therapeutic Mechanisms of NSCs in GB

**Fig. 4:**
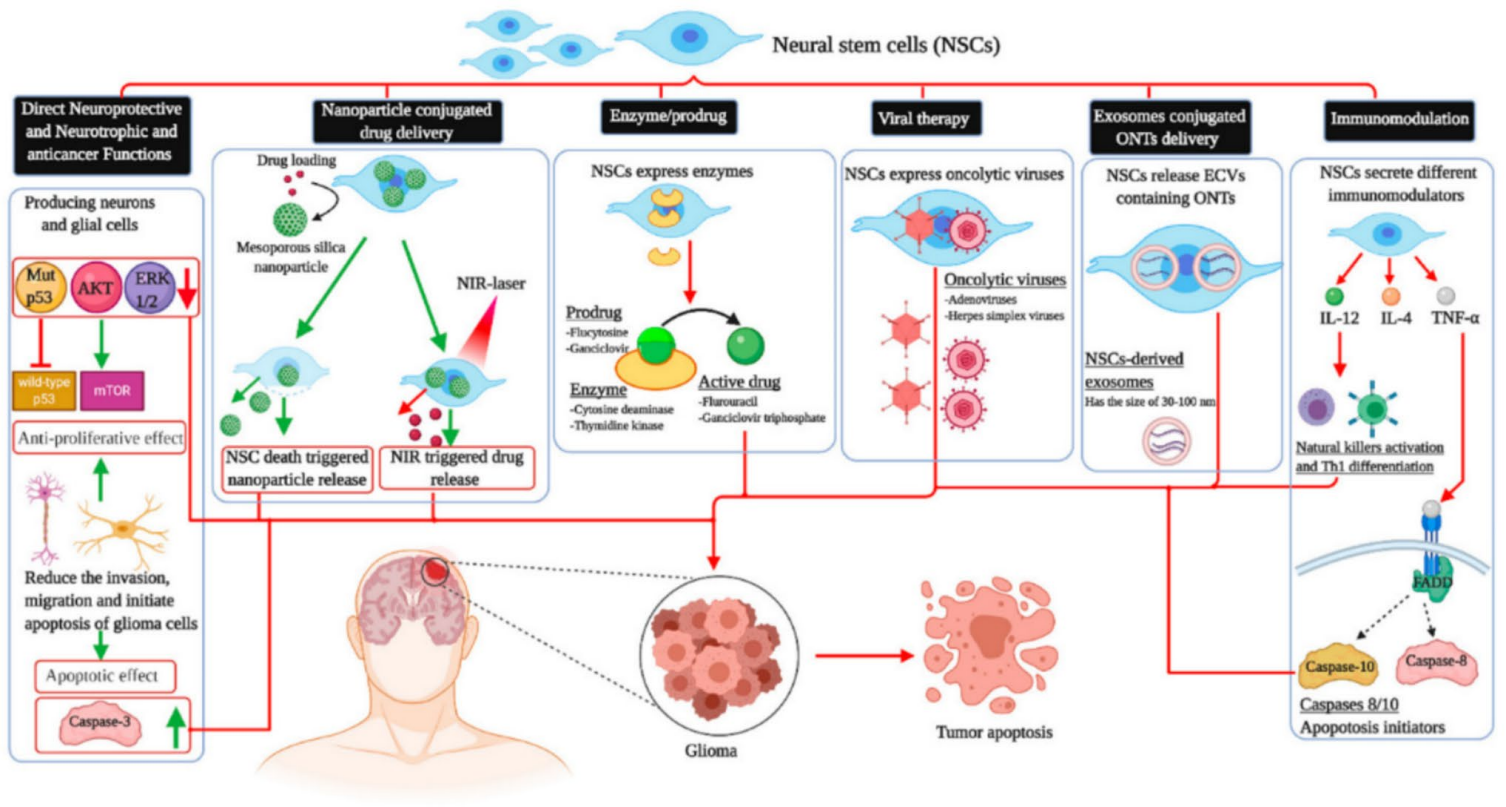
Therapeutic applications of NSCs in GB treatment. NSCs have been utilized to induce GB cell death through a variety of molecular and immunological mechanisms. Key pathways and mediators include protein kinase B (AKT), extracellular signal-regulated kinases 1 and 2 (ERK1/2), tumor necrosis factor-alpha (TNF-α), interleukins IL-4 and IL-12, and Fas-associated death domain protein (FADD). Additional strategies involve oligonucleotide therapeutics (ONTs), activation of T helper 1 (Th1) responses, modulation of the mammalian target of rapamycin (mTOR) pathway, near-infrared (NIR) responsive systems, and delivery via extracellular vesicles (ECVs) (Benmelouka et al. 2021)

NSCs offer multiple therapeutic strategies for GB treatment due to their tumor-targeting properties, adaptability, and natural ability to migrate selectively toward tumor cells alongside their unique capability to bypass the BBB and infiltrate invasive tumor margins, enabling the targeted delivery of therapeutic agents directly into the TME (Chia et al. 2020). The main therapeutic applications of NSCs include direct neuroprotective and anticancer effects, nanoparticle-conjugated drug delivery, enzyme/prodrug therapy, viral therapy, exosome-mediated oligonucleotide delivery, and immunomodulation (Fig. 4).

In patients with glioblastoma multiforme (GBM) or other gliomas, endogenous neural stem cells (NSCs) may harbor oncogenic mutations due to the tumor’s local and systemic genetic influences. This raises safety concerns regarding the use of autologous NSCs for therapeutic purposes. Clarifying the potential sources of therapeutic NSCs is therefore essential. These sources may include allogeneic NSCs from healthy donors, fetal-derived NSCs, induced pluripotent stem cell (iPSC)-derived NSCs, or directly induced NSCs from somatic cells. Each option carries distinct ethical, immunological, and safety considerations. Before clinical application, therapeutic NSCs undergo rigorous quality control, including genomic profiling to detect oncogenic mutations, karyotyping to assess chromosomal stability, and in vivo tumorigenicity assays to exclude malignant transformation risk. Such preclinical screening is crucial to ensure both the genetic stability and functional integrity of NSCs intended for glioma therapy (Calinescu et al. 2021; Mahdi et al. 2025).

NSCs exert direct anti-tumor effects by modulating pathways such as p53, protein kinase B (AKT), extracellular signal-regulated kinases 1 and 2 (ERK) 1/2, and the mammalian target of rapamycin (mTOR), leading to apoptosis via caspase-3. NSCs can also deliver nanoparticles loaded with chemotherapeutics, triggered by cell death or near-infrared (NIR) stimulation. In enzyme/prodrug therapy, engineered NSCs express enzymes that convert non-toxic prodrugs into active anticancer agents. NSCs can also secrete OVs to selectively infect tumor cells. Additionally, NSC-derived exosomes deliver oligonucleotide therapeutics (ONTs) directly to tumors which are capable of modulating gene expression through diverse mechanisms, including RNA interference (RNAi), RNase H-mediated transcript degradation, splicing modulation, inhibition of non-coding RNAs, gene activation, and precise genome editing. In immunotherapy, NSCs release cytokines like interleukin-12 (IL-12), interleukin-4 (IL-4), and tumor necrosis factor-alpha (TNF-α) to activate immune responses and induce apoptosis via Fas-associated death domain protein (FADD) and caspase-8/10 pathways. These approaches highlight the diverse and promising roles of NSCs in GB therapy (Benmelouka et al. 2021; Luzzi et al. 2020; Ramanathan and Lorimer 2022; Roberts et al. 2020).

In addition, NSCs possess a unique ability to migrate toward glioma lesions, making them promising delivery vehicles for therapeutic agents. This tumor tropism is primarily mediated by chemotactic cues within the tumor microenvironment (TME). Chemokine–receptor interactions, such as the CXCL12/CXCR4 and HGF/c-Met axes, play central roles in guiding NSCs to tumor sites. Additional factors including vascular endothelial growth factor (VEGF), platelet-derived growth factor (PDGF), inflammatory cytokines, and extracellular matrix remodeling further facilitate NSC infiltration into tumor tissue. Hypoxia within the tumor niche enhances the expression of migratory receptors on NSCs, reinforcing this homing effect. Understanding these molecular and microenvironmental mechanisms not only explains the natural tumor-seeking behavior of NSCs but also informs strategies to enhance their targeting precision, such as receptor overexpression or chemokine preconditioning, to improve therapeutic delivery and efficacy (Emami Nejad et al. 2021; Mahdi et al. 2025; Namba et al. 2015; Zhang et al. 2021).

Collectively, these strategies, ranging from gene modulation and immunotherapy to exosome-mediated interventions, are currently being evaluated in preclinical models and early-phase clinical trials with preliminary findings which suggest that NSC-based therapies hold considerable potential to improve targeting, minimize systemic toxicity, and ultimately enhance outcomes for patients with GB (Y. Liu et al. 2024; Lutfi Ismaeel et al. 2023).

### Engineered NSC Vectors

While the therapeutic potential of NSC therapy has been significantly demonstrated in animal models, several challenges remain that must be addressed to improve tissue regeneration outcomes. A primary limitation is the low survival rate of transplanted NSCs, which reduces their therapeutic efficacy in-vivo. In addition to the insufficient migration of these cells to the target site and their poor integration into the host system. This may be attributed to the tumor microenvironment, characterized by elevated reactive oxygen species (ROS) and inflammatory mediators, coupled with inadequate signaling for proper differentiation and integration (Baklaushev et al. 2019; Wei et al. 2024).

To address the limitations associated with NSC-based therapies, genetic modification strategies have been developed to enhance their therapeutic efficacy. Such modifications aim to improve NSC survival, migration, and differentiation within the TME, thereby optimizing their functional contribution following (Benmelouka et al. 2021; Lutfi Ismaeel et al. 2023). Overexpression of neurotrophic factors, including brain-derived neurotrophic factor (BDNF) and glial cell line-derived neurotrophic factor (GDNF), activates pro-survival signaling pathways such as PI3K/Akt and MAPK/ERK, increasing cell persistence in vivo (Matsuzaka and Yashiro 2025; Numakawa and Kajihara 2023). Similarly, engineering NSCs to express higher levels of chemokine receptors, such as CXCR4 and c-Met, augments their responsiveness to tumor-derived chemotactic signals (e.g., CXCL12, hepatocyte growth factor), thereby enhancing tumor tropism (Kumar et al. 2024). In addition, genetic modulation can extend to microRNAs (miRNAs), enabling fine-tuned regulation of gene networks to promote lineage-specific differentiation or upregulate therapeutic mediator production. Collectively, these engineering approaches provide a robust mechanistic basis for improving NSC functionality in glioma models and inform the development of next-generation cell-based therapies (Kumar et al. 2020).

### Oncolytic Virus Delivery

Oncolytic virotherapy has emerged as a promising anticancer strategy that utilizes naturally occurring or genetically modified viruses to selectively target and eliminate glioma cells. Various OVs have demonstrated the capacity to infect glioma cells by inducing apoptosis or stimulating robust anti-tumor immune responses (Asija et al. 2023). OVs are particularly suitable for GB therapy owing to several intrinsic advantages such as their compatibility with the brain microenvironment, the non-metastatic nature of GB outside the CNS, and the rapid proliferation of tumor cells, which promotes efficient viral replication (Hamad et al. 2023).

**Fig. 5:**
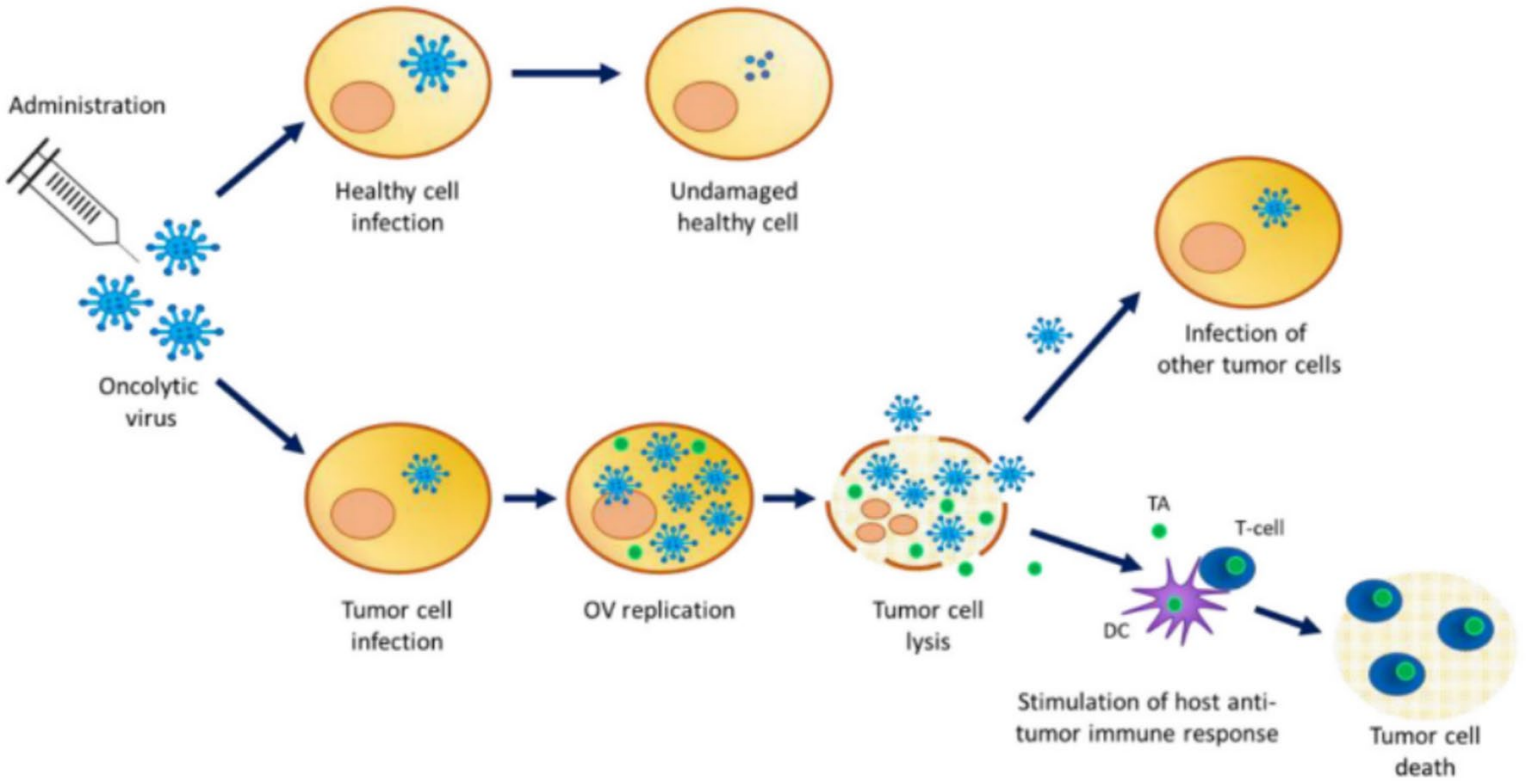
Presents the mechanism of Action of Oncolytic Viruses: Oncolytic viruses (OVs) can infect normal cells; however, they are typically unable to replicate within them. In contrast, upon infecting cancer cells, OVs efficiently replicate and assemble new viral particles. The accumulation of a high viral load within tumor cells ultimately leads to cellular lysis, releasing both progeny viral particles and tumor-associated antigens (TAAs) into the tumor microenvironment (TME). The newly produced viral particles can then infect adjacent tumor cells, perpetuating the oncolytic cycle. Simultaneously, the release of tumor antigens promotes activation of the host’s anti-tumor immune response, with dendritic cells (DCs) playing a crucial role in antigen presentation and subsequent immune activation. (Onnockx et al. 2023)

OVs are broadly classified into two categories: replication-competent and selectively replication-competent viruses, as illustrated in Fig. 5. Replication-competent viruses directly infect and replicate within tumor cells, leading to their lysis and the subsequent spread of viral progeny to neighboring malignant cells. This mechanism exerts anti-tumor effects through two complementary pathways: direct oncolysis and the activation of an immune response that transforms immunologically "cold" tumors into "hot" tumors, thereby enhancing immune system engagement (Onnockx et al. 2023). Conversely, selectively replication-competent viruses serve primarily as platforms for gene therapy. They act as viral vectors that deliver therapeutic genes into tumor cells, where gene expression triggers anti-tumor effects without extensive viral replication (Estevez-Ordonez et al. 2021).

Following tumor cell lysis, DCs are recruited to the TME, where they detect pathogen-associated molecular patterns (PAMPs) derived from the OV and damage-associated molecular patterns (DAMPs) from the lysed tumor cells. These DCs then process and present tumor-associated antigens (TAAs) to CD4⁺ T cells within regional lymph nodes, initiating T cell activation. Activated CD4⁺ T cells promote the differentiation of TAA-specific cytotoxic CD8⁺ T cells, which subsequently migrate back to the tumor site to orchestrate the targeted killing of residual malignant cells (Shoaf and Desjardins 2022).

### Exosome-Based Delivery

Extracellular vesicles (EVs) are membrane-bound particles secreted by both prokaryotic and eukaryotic cells, either as part of normal physiological processes or in response to pathological conditions. These vesicles are integral to intercellular communication, mediating the transport of bioactive molecules such as proteins, lipids, and nucleic acids which regulate diverse cellular functions. Among the various types of EVs, the most studied are exosomes, microvesicles, and apoptotic bodies. Exosomes originate from the endosomal system through a well-defined process of biogenesis, intracellular trafficking, and exocytosis and their size typically ranges from 40 to 150 nm (Kalluri and LeBleu 2020; Patel et al. 2023). They are structurally characterized by a lipid bilayer therefore, they are capable of carrying a wide spectrum of molecular cargo, including enzymes, transcription factors, DNA fragments, mRNAs, miRNAs, and long non-coding RNAs (lncRNAs) (Thakur et al. 2022). They are secreted by numerous cell types, such as tumor cells, lymphocytes, DCs, adipocytes, and fibroblasts, and have been identified in various biological fluids including blood, plasma, cerebrospinal fluid (CSF), urine, saliva, milk, amniotic fluid, synovial fluid, and malignant ascites (Paskeh et al. 2022).

**Fig. 6:**
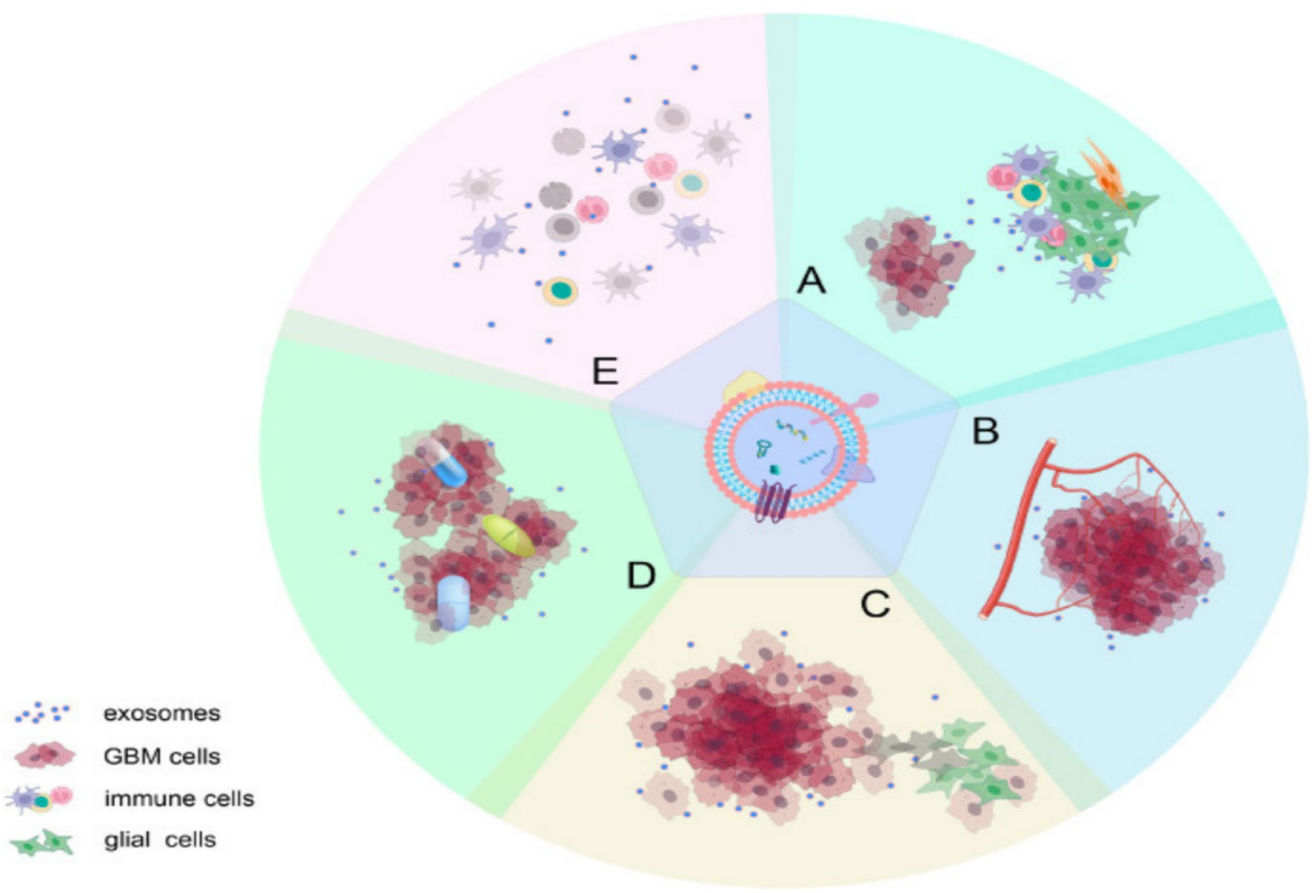
The roles of glioma-derived exosomes in glioma progression include: **A** inducing alterations in the tumor microenvironment; **B** mediating angiogenesis; **C** influencing the proliferation and invasiveness of glioma cells; **D** contributing to drug resistance; and **E** suppressing immune responses. It is crucial to recognize that the effects of active molecules on tumor progression are typically not confined to a single process but instead exert broad influence across multiple facets of glioma development. (Yang et al. 2024)

Exosomes are nanoscale vesicles, distinguished by their ability to effectively traverse the BBB, thereby serving as efficient carriers for therapeutic delivery (Rehman et al. 2023). In addition to their transport capabilities, exosomes significantly contribute to glioma progression by facilitating the transfer of biological molecules and enhancing intercellular communication within the TME. These properties underscore their potential to advance glioma diagnosis, prognosis, and treatment (Fig. 6) (Daßler-Plenker et al. 2020; Yang et al. 2024).

## Neural Stem Cells in Glioblastoma: Therapeutic Potential and Clinical Trials

### Preclinical Studies

A study by (Batalla-Covello et al. 2021) sought to test the hypothesis of packaging CRAd-S-pk7 which is an OV within NSCs (NSC-CRAd-S-pk7) for the protection of the virus from immune-mediated destruction which would allow effective delivery to tumor sites. Hence, this study was conducted with the aim of evaluation of the therapeutic potential and safety of multiple administrations of NSC-CRAd-S-pk7, while addressing concerns related to viral inactivation by neutralizing antibodies. This study provided evidence that NSCs effectively protect CRAd-S-pk7 from complement-mediated neutralization, thereby preserving viral infectivity in both in-vitro and in-vivo settings. Encapsulation within NSCs enabled sustained intratumoral viral presence across multiple administrations, despite the development of anti-adenoviral antibodies. In addition, long-term survival analyses in immunocompetent mouse models demonstrated that three weekly intracerebral administrations of NSC-CRAd-S-pk7 significantly improved survival compared to a single administration, without inducing systemic toxicity, neurological impairment, or significant weight loss. Whereas immunological profiling indicated that repeated dosing elicited enhanced anti-tumor immune responses, characterized by increased infiltration of CD3 + and CD8 + T cells into the TME and a concomitant reduction in PD-1 expression, highlighting the importance of the adaptive immune system in mediating therapeutic benefit. Importantly, no evidence of anti-NSC immune responses was observed following multiple administrations. Nevertheless, the study has several limitations, since it was confined to a dose equivalent to 150 million NSC-CRAd-S-pk7s, thus, disqualifying assessment of potential dose-dependent effects and the optimal frequency and long-term consequences of repeated administration were not systematically evaluated. Furthermore, although murine models provide valuable insights, species-specific differences in immune responses may limit the direct extrapolation of these findings to human clinical settings. Despite these limitations, the data strongly support the clinical development of multiple intracerebral NSC-CRAd-S-pk7 administrations as a strategy to enhance the efficacy of oncolytic virotherapy in GB.

**Fig. 7:**
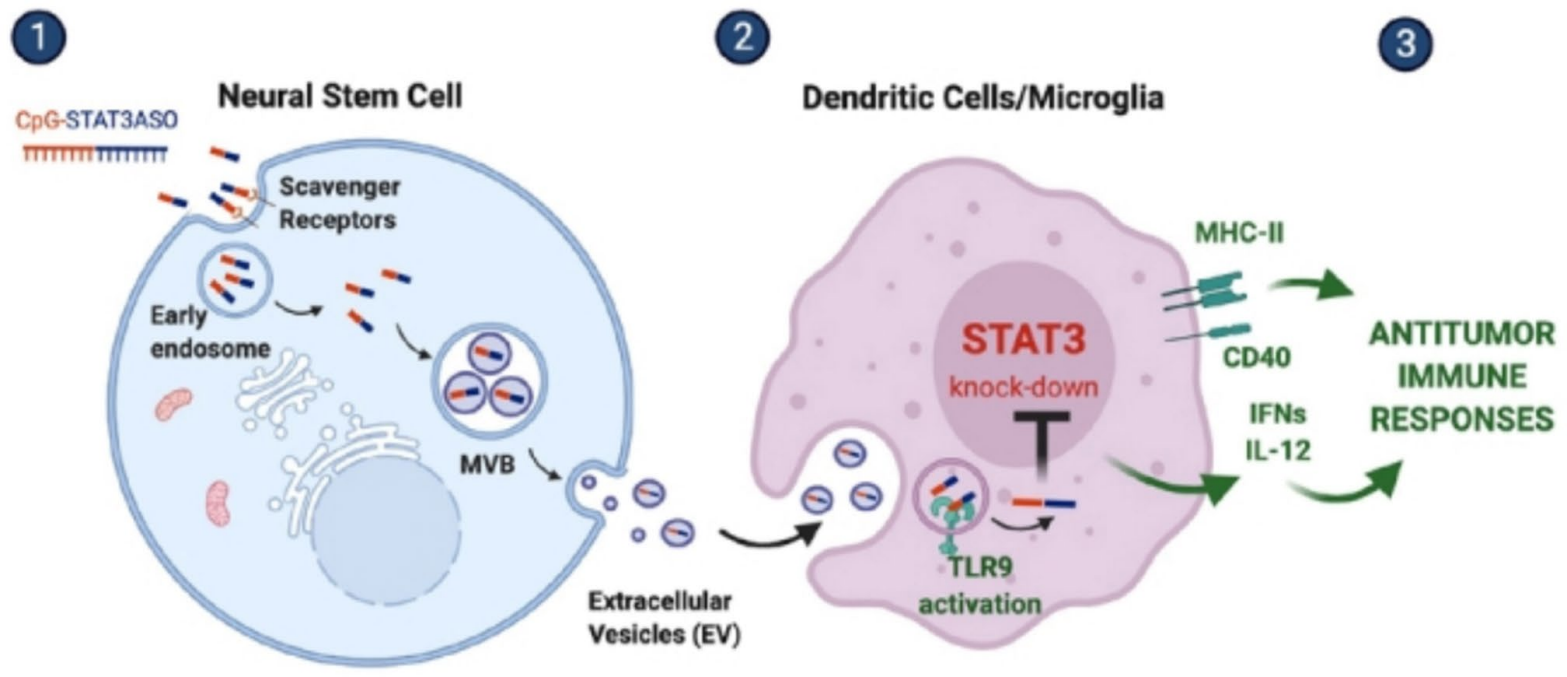
Neural stem cell (NSC)-derived extracellular vesicles (EVs) modulate anti-tumor immune responses in GB via STAT3 knockdown and TLR9 activation. (1) NSCs loaded with CpG-STAT3ASO (a STAT3-targeting antisense oligonucleotide coupled to CpG) release EVs containing scavenger receptors and therapeutic cargo. These EVs are internalized by dendritic cells/microglia via early endosomes and multivesicular bodies (MVBs). (2) EV-delivered CpG-STAT3ASO simultaneously knocks down STAT3 (an immunosuppressive signal) and activates TLR9, promoting dendritic cell maturation (MHC-II, CD40) and pro-inflammatory cytokine secretion (IFNs, IL-12). (3) This dual action primes anti-tumor T cell responses. (Adamus et al. 2021)

Another study by (Adamus et al. 2021) evaluated the possibility of utilizing clinically tested NSCs18 and exosomes derived from NSCs as delivery vehicles for ONTs into the glioma microenvironment. This study demonstrates that NSCs can spontaneously internalize and encapsulate cytosine-phosphate-guanine (CpG)-conjugated signal transducer and activator of transcription 3 (STAT3) antisense oligonucleotides (CpG-STAT3ASO) into exosomes, enabling efficient and dose-dependent therapeutic cargo loading. These exosomes retained and enhanced the immunostimulatory potential of CpG-STAT3ASO, as shown by increased IL-12 expression and NF-κB activation in human DCs and macrophages, alongside effective STAT3 downregulation. Importantly, NSC-derived exosomes delivered CpG-STAT3ASO to glioma-associated myeloid cells in-vivo, significantly upregulating activation markers and reducing tumor growth in subcutaneous glioma models (Fig. 7). However, several limitations were mentioned including short circulatory half-life and potential inflammatory risks associated with ONTs, infeasibility of repeated intracranial NSC injections in mice, unclear mechanisms behind the enhanced immune-stimulation by exosomes encapsulated CpG-STAT3ASO, and the possible immunosuppressive influence of native NSCs and their exosomes. Additionally, optimizing dosing regimens and verifying clinical translatability remain essential next steps. Despite these challenges, the study offers a compelling framework for the development of NSCs-based delivery systems for oligonucleotide immunotherapies targeting immune-evasive tumors such as GB.

**Fig. 8:**
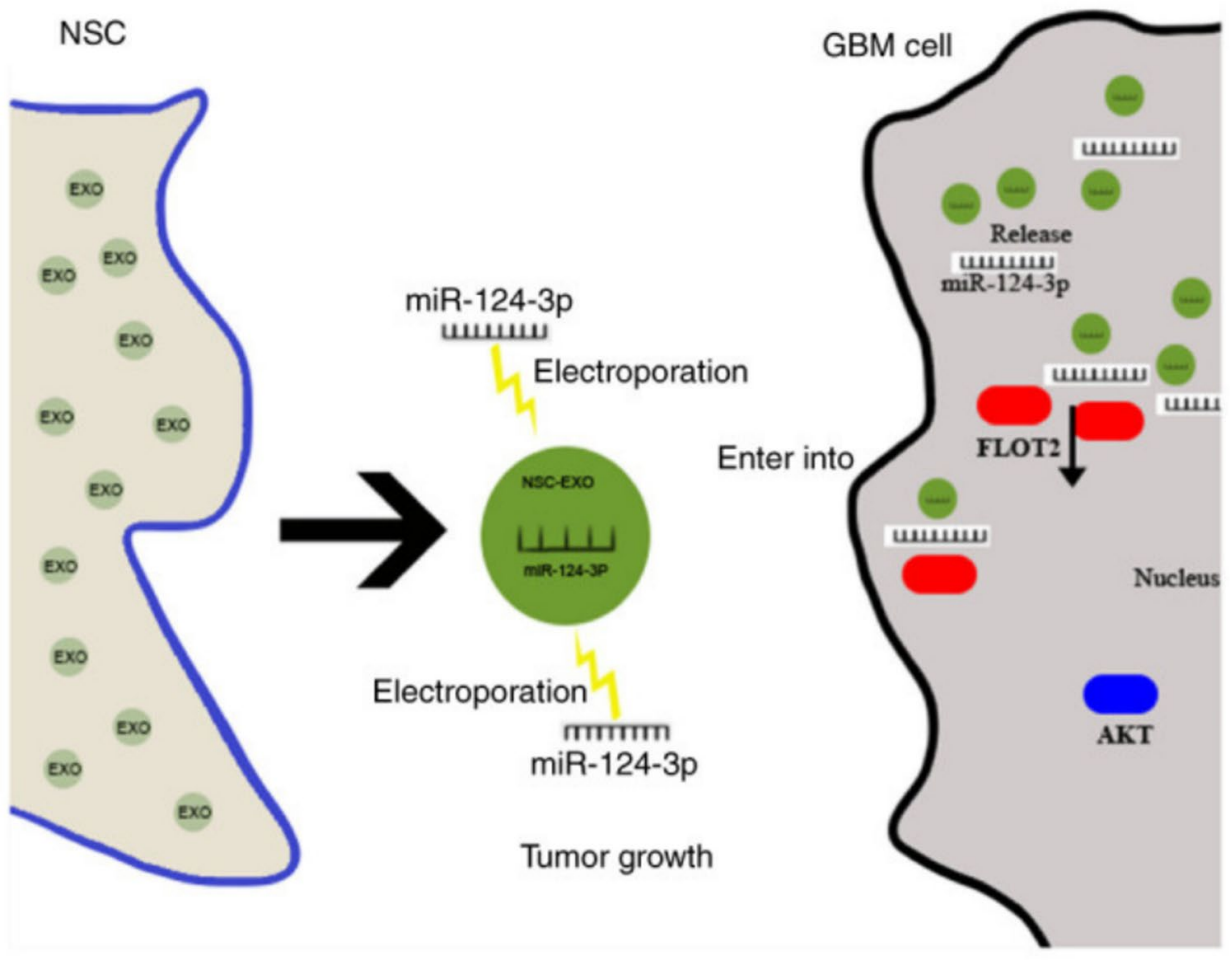
A schematic representation illustrating the transfer of miR-124-3p by NSC-derived exosomes (NSC-EXOs) to inhibit glioma growth through targeting FLOT2. Exosomes from NSCs were loaded with miR-124-3p mimics via electroporation. Upon delivery to glioma cells, the miR-124-3p was released by the exosomes and specifically bound to the 3'-untranslated region of FLOT2 mRNA, leading to the suppression of FLOT2 expression. This, in turn, resulted in the downregulation of the AKT1 signaling pathway, ultimately inhibiting glioma cell proliferation. Abbreviations: NSC, neural stem cell; EXOs, exosomes; FLOT2, flotillin 2 (Qian Cheng et al. 2022)

Another recent study by (Qian Cheng et al. 2022) explored the therapeutic efficacy of neural stem cell-derived exosomes (NSC-EXOs) loaded with miR-124-3p which is a tumor suppressor, as a potential treatment for GB. The findings demonstrated that NSC-EXOs, when labeled and introduced into glioma cells, effectively delivered miR-124-3p into both the cytoplasm and nucleus, resulting in a marked increase in miR-124-3p expression. This cargo transfer led to a significant reduction in glioma cell proliferation, invasion, and migration, as evidenced by multiple in-vitro assays. Further analysis identified flotillin 2 (FLOT2) as a key target of miR-124-3p, with the exosome-mediated delivery of miR-124-3p resulting in the downregulation of FLOT2 expression. The inhibition of FLOT2 expression subsequently suppressed glioma progression via the FLOT2/AKT1 signaling pathway. In-vivo studies reinforced these findings, showing that NSC-EXOs loaded with miR-124-3p significantly inhibited tumor growth in mouse models, with no observed toxicity. Molecular analyses of tumor tissues confirmed a marked reduction in both FLOT2 and AKT1 expressions in the treated group (Fig. 8). Furthermore, bioinformatics analyses revealed that high FLOT2 expression is associated with poor prognosis in GB patients. Nevertheless, several limitations are present in the current study. Notably, the potential impact of the BBB on the delivery of NSC-EXOs was not investigated. While EXO-miR-124-3p therapy has demonstrated encouraging outcomes, it is probable that multiple molecular targets are involved, necessitating further identification and characterization. Additionally, the absence of an in situ xenograft model of GB limits the ability to accurately replicate the TME. Moreover, the precise components of the exosomes that mediate the regulation of glioma cells remain unclear and require further exploration.

A study conducted by (Vaidya et al. 2023) aimed to investigate the role of paracrine signaling (particularly via EVs and diffusible macromolecules) in the reciprocal interaction between NSCs and GB cells, using a non-contact co-culture model. By allowing the two cell types to share culture media without physical contact, the study aims to elucidate how NSC- and GB-derived factors influence each other’s behavior and contribute to cancer stem cell (CSC) formation, thereby identifying molecular targets that could be leveraged to improve GB therapy. The findings of this study demonstrated that GB cells and NSCs can internalize each other’s EVs in a non-contact co-culture model, highlighting the importance of EV-mediated communication in altering cellular behavior. GB cells exposed to NSC-derived factors exhibited upregulation of several stemness- and drug resistance-related genes, including CD133, CD44, CD9, SOX9, TUBB3, MGMT, and ABCG2, suggesting a shift toward a CSC-like phenotype. In contrast, NSCs exposed to GB-derived factors showed downregulation or no change in similar markers, implying a potential differentiation effect. Interestingly, embryonic stem cell genes like SOX2 and NANOGP8 remained unaltered, indicating that the initial CSC transformation may be driven by neural progenitor markers rather than classical embryonic stemness genes. These findings suggest that factors secreted by NSCs can influence the transcriptional profile of GB cells, promoting CSC characteristics, while GBM-derived signals may suppress NSC stemness. However, limitations of this study include limited the duration of exposure, the co-culture system does not fully replicate the complexity of the TME, and gene expression was assessed at a single time point, potentially missing dynamic transcriptional changes. Moreover, while EV uptake was confirmed, the specific EV cargo responsible for the observed effects was not characterized, and the influence of other diffusible factors cannot be excluded. These limitations highlight the need for further in-vivo and longitudinal studies to clarify the molecular mechanisms driving CSC induction in GB.

**Fig. 9:**
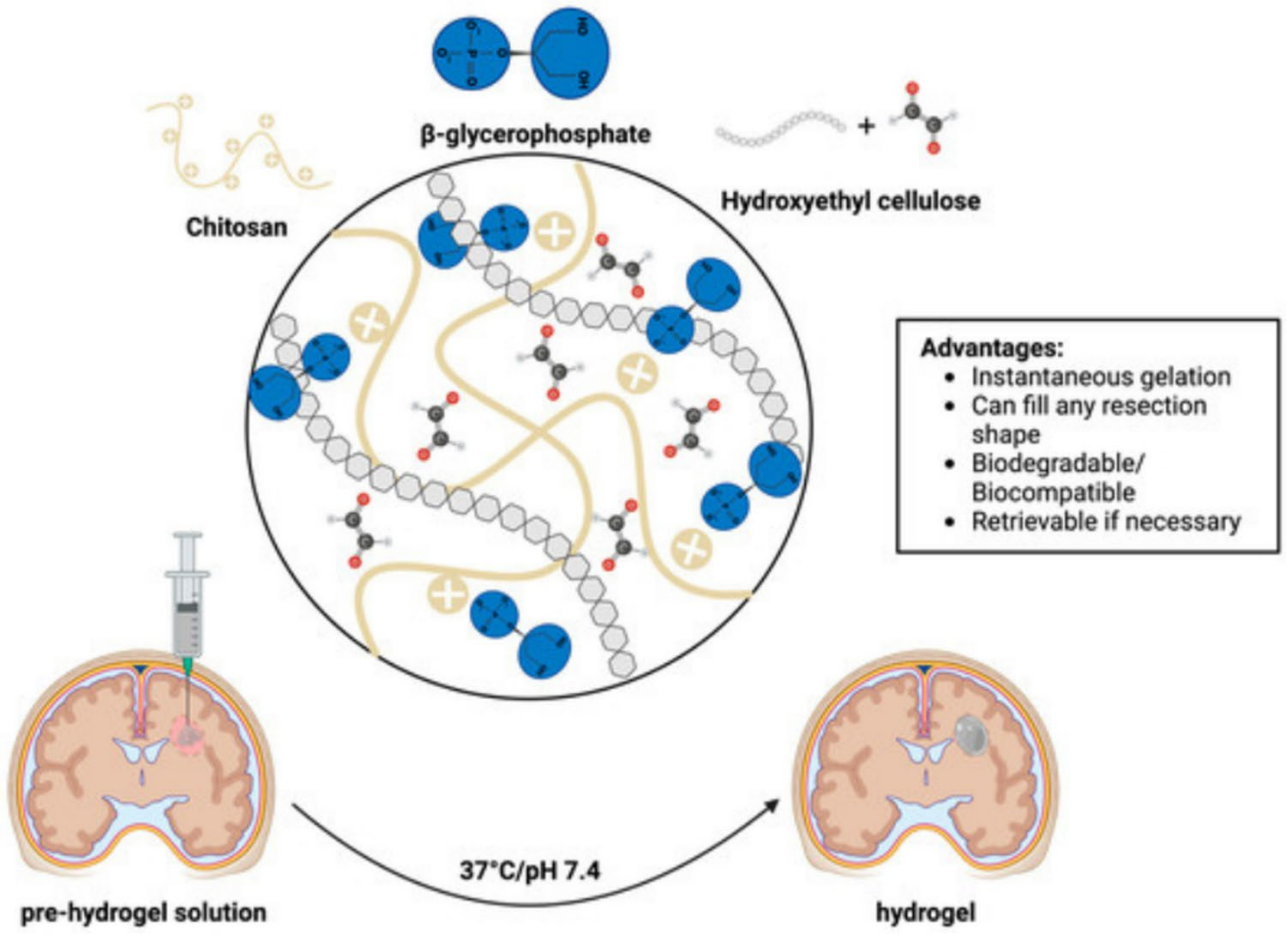
The CS hydrogel demonstrated (1) rapid gelation kinetics under physiological conditions and (2) superior biodegradability and cytocompatibility. Furthermore, this hydrogel system offers the ability to (1) conform to any resection shape and (2) be retrieved if required. (King et al. 2025)

A more recent study was conducted with the aim of evaluating the safety, therapeutic efficacy, and persistence of an injectable, in situ forming chitosan-based (CS) hydrogel scaffold as a delivery platform for tumoricidal induced neural stem cells (iNSCs) in a post-surgical GB model. Specifically, the study seeks to determine whether the CS hydrogel can enhance iNSC retention in the surgical cavity, prolong survival in U87-MG GB xenograft models, and serve as a promising adjunct therapy for resectable GB. The study demonstrates that CS-based injectable hydrogels significantly enhance the retention and persistence of iNSCs in a GB resection cavity, improving the effectiveness of post-surgical therapy. The CS hydrogel demonstrated fast gelation kinetics, biodegradability, and cytocompatibility, allowing it to effectively encapsulate iNSCs and prolong their persistence by up to 9.3-fold compared to direct cell injection (Fig. 9). This extended retention led to a marked reduction in GB tumor volumes and a 50% increase in median survival in the treated animals. Additionally, the hydrogel showed minimal adverse tissue reactions, such as mild edema and gliosis, confirming its safety. However, limitations include variability in surgical resections, which affected the consistency of tumor removal and may have influenced the therapeutic outcomes. Furthermore, the study lacks detailed analysis of the impact of CS hydrogel encapsulation on iNSC gene expression, migration, and differentiation, which could impact the efficacy of tumor killing (Table 1). The current results are promising, but further investigation into more clinically relevant GB models and large animal studies is required to confirm the translatability of this technology (King et al. 2025).

**Table 1:** Representative preclinical studies demonstrating neural stem cells’ tumor-tropic properties and therapeutic applications in glioblastoma models

Model	Intervention design	Key findings	References
Immunocompetent mouse GBM	NSCs carrying CRAd-S-pk7 OV	Enhanced viral persistence, improved survival	Batalla-Covello et al. (2021)
Human glioma xenografts	NSC-derived exosomes with CpG-STAT3ASO	STAT3 knockdown, ↓tumor growth	Adamus et al. (2021)
GBM cell lines and mice	NSC-EXOs with miR-124-3p	↓Proliferation/migration	Qian Cheng et al. (2022)
Non-contact co-culture of NSCs and GB cells	Paracrine signaling via EVs and diffusible macromolecules	Reciprocal EV uptake; GB cells exposed to NSC-derived factors ↑CSC/stemness genes (CD133, CD44, CD9, SOX9, TUBB3, MGMT, ABCG2); NSCs exposed to GB factors ↓stemness; embryonic stem cell markers unchanged; highlights EV-mediated CSC induction; limitations: short exposure, simplified TME, single time-point gene analysis, uncharacterized EV cargo	Vaidya et al. (2023)
Post-surgical GBM model	iNSCs in chitosan hydrogel	↑Cell retention, ↑survival	King et al. (2025)

### Recent Clinical Trials

A phase I clinical trial explored the safety, side effects, and optimal dosing of genetically modified NSCs combined with flucytosine and leucovorin for treating recurrent high-grade gliomas. The NSCs used in the study were engineered to express the enzyme cytosine deaminase (CD), which converts the prodrug flucytosine (5-FC) into 5-fluorouracil (5-FU), a chemotherapeutic agent that selectively targets and destroys rapidly dividing tumor cells while sparing healthy brain tissue. The NSCs were administered directly into the brain, where they migrate toward tumor sites. After allowing time for the NSCs to spread, participants underwent a 7-day course of oral 5-FC, potentially accompanied by leucovorin to enhance the effectiveness of 5-FU. To maintain treatment continuity, a Rickham catheter, placed during surgery, delivering additional NSC doses every two weeks, followed by further rounds of 5-FC treatment. The primary objectives of this trial are to determine the phase II recommended dose and evaluate the feasibility of multiple NSC administrations. Secondary objectives include assessing the development of immune responses against the NSCs, characterizing the relationship between drug concentrations in the brain and systemically, determining clinical benefits, and analyzing the fate of NSCs during autopsy. This innovative treatment strategy, leveraging NSCs for targeted drug delivery, offers a promising approach to addressing high-grade gliomas (NCT02015819, 2021).

Another open-label, phase I, dose-escalation clinical trial employing a traditional 3 + 3 study conducted by (Fares et al. 2021) aimed to assess the safety and therapeutic potential of NSC-CRAd-S-pk7 which is an engineered oncolytic adenovirus carried by NSCs in 12 individuals newly diagnosed with high-grade glioma (GB and anaplastic astrocytoma). This phase 1 clinical trial demonstrated that NSC-CRAd-S-pk7 is safe and feasible for patients with newly diagnosed high-grade gliomas. Injection into the resection cavity during surgery, followed by standard chemo-radiotherapy, caused no dose-limiting toxicity, and treatment-related complications were rare and manageable. Since no formal dose-limiting toxicity was identified, the highest tested dose was 1.50 × 10⁸ NSCs carrying 1.875 × 10^11^ viral particles is recommended for evaluation in phase 2 trials. Additionally, multiple injections enhanced viral distribution, leveraging the tumor-tropic nature of NSCs without delaying postoperative therapies. Immune analyses revealed increased CD8⁺ T cell activation and cytokine production, suggesting systemic immune engagement. Histopathological findings supported therapeutic efficacy, showing reduced expression of survivin and syndecan-1. No long-term presence of NSCs or viral particles was detected, affirming treatment safety. The findings also indicated increased PD-1 expression, supporting future exploration of combining NSC-CRAd-S-pk7 with immune checkpoint inhibitors. However, limitations such as the single-arm, open-label design and small sample size underscore the need for larger, controlled studies to validate these findings.

A more recent ongoing phase I clinical trial investigates the safety and potential efficacy of multiple intracerebral doses of NSC-CRAd-S-pk7, a neural stem cell-based oncolytic virotherapy, in patients with recurrent high-grade gliomas. The primary objective is to determine the maximum tolerated number of treatment cycles (MTC) based on dose-limiting toxicities, overall safety profile, and preliminary therapeutic activity, thereby informing future Phase II studies. Secondary objectives include evaluating immune responses against the NSCs and viruses, assessing intracerebral biodistribution and possible migration outside the brain, and monitoring the presence of viral particles in CSF and blood. The study also aims to estimate disease response rates, progression-free survival at six months, and overall survival at nine months. Additionally, it explores changes in tumor biomarkers (HSPG and survivin) and investigates immune landscape alterations in the TME pre- and post-treatment. Finally, a bio-mathematical model will be developed to predict tumor response dynamics based on MRI imaging (Table 2). Patients included in this trial will undergo standard-of-care surgical resection followed by weekly intracerebral administration of NSC-CRAd-S-pk7 for up to four doses, with longitudinal follow-up at defined intervals to assess outcomes (Portnow et al. 2024).

**Table 2:** Clinical trials evaluating neural stem cell-based therapies for glioblastoma, highlighting safety, feasibility, and tumor-targeting capacity

Trial/NCT No	Phase	Patient population	Key findings
NCT02015819 (2021)	I	Newly diagnosed GB	Feasible, well tolerated; evidence of local NSC distribution
Portnow et al. (2024)	I/II	Recurrent GB	Preliminary results show prolonged survival in some patients
Fares et al. (2021)	I	Recurrent GB	Safe; preliminary efficacy in tumor volume reduction

## Limitations and Future Perspectives

Overall, NSCs offer several distinct advantages over conventional therapies in the treatment of GB, primarily due to their unique biological properties and therapeutic versatility. One of the most critical features of NSCs is their inherent tumor-tropic behavior, which enables them to migrate selectively toward tumor sites and deliver therapeutic agents directly to the GB microenvironment. This ability also allows NSCs to cross the BBB, a major obstacle for traditional chemotherapeutic agents such as paclitaxel and temozolomide. In addition to their tumor-homing capabilities, NSCs can be engineered to carry and release a range of therapeutic cargos including oncolytic viruses, prodrug-converting enzymes, and oligonucleotides, ensuring localized drug delivery while minimizing systemic toxicity. For instance, NSC-CRAd-S-pk7 has been shown to enhance oncolytic virus persistence and shield the virus from immune clearance.

NSCs also exert multimodal mechanisms of action: they can mediate tumor cell lysis through oncolytic virus delivery, silence oncogenes via exosomes carrying miRNAs or antisense oligonucleotides (e.g., miR-124-3p, STAT3 antisense), modulate the immune response through cytokine secretion (e.g., IL-12, TNF-α), and activate chemotherapeutic agents in situ through enzyme/prodrug systems. Importantly, NSC-based therapies restrict cytotoxic exposure to healthy tissues, reducing systemic toxicity compared to agents like lomustine or temozolomide. Furthermore, they can effectively target hypoxic regions within the tumor and bypass mechanisms of chemoresistance that limit the efficacy of conventional treatments. NSCs also complement existing modalities such as surgery, radiotherapy, and chemotherapy, functioning as adjuncts to improve local tumor control and potentially delay recurrence. Finally, NSCs are highly adaptable and can be genetically modified to enhance their survival, migratory capacity, and therapeutic function, enabling personalized treatment strategies tailored to individual tumor profiles.

Despite the promising potential of NSCs in the treatment of GB, several limitations remain, both in the preclinical and clinical application of NSC-based therapies. One of the primary challenges is the heterogeneous nature of GB itself. GB tumors exhibit vast genetic and molecular diversity, leading to variability in response to treatment. This tumor heterogeneity not only complicates the prediction of treatment outcomes but also increases the likelihood of resistance mechanisms (Becker et al. 2021; Rabah et al. 2023). Furthermore, although NSCs have demonstrated the ability to bypass the BBB, their targeting efficiency remains suboptimal, highlighting the need for further optimization to improve therapeutic delivery (Zhang 2024).

Moreover, the safety of NSC-based therapies remains a significant concern. While preclinical studies support their potential to deliver therapeutic agents and modulate the TME, risks such as tumorigenicity and uncontrolled differentiation persist. NSCs may inadvertently promote tumor growth or form secondary malignancies. Additionally, emerging evidence suggests that NSCs could trigger unintended immune responses or contribute to a supportive TME, potentially facilitating tumor progression (Fontán-Lozano et al. 2020; Xu et al. 2021).

From a clinical perspective, translating NSC-based therapies from animal models to human patients has proven challenging. Many promising preclinical results have failed to translate effectively in clinical trials, partly due to fundamental differences in the TME between animal models and human patients. Moreover, patient-specific variables such as immune system status, age, and tumor subtype can markedly influence the therapeutic efficacy of NSC-based interventions (Boccellato and Rehm 2022). NSC therapies are often administered alongside standard treatments, including surgery, radiotherapy, and chemotherapy; however, the interactions between these modalities and NSC behavior remain poorly understood. This complexity underscores the need for a comprehensive, integrative approach to evaluating NSC-based therapies within the framework of multimodal treatment strategies (Benmelouka et al. 2021).

Another key limitation in the current body of research is the lack of systematic evaluation of how different glioma subtypes respond to NSC–based therapies. While gliomas encompass a broad spectrum of histological and molecular profiles ranging from GBM to oligodendroglioma, most preclinical and clinical studies have focused primarily on GBM, often without stratifying results according to tumor grade, genetic alterations (e.g., IDH mutation status, 1p/19q codeletion), or molecular phenotype. This gap in knowledge makes it unclear whether variations in tumor biology influence NSC homing efficiency, survival, or therapeutic payload delivery. Given that tumor microenvironmental factors such as chemokine gradients, extracellular matrix composition, and immune infiltration can differ significantly between subtypes, it is plausible that these differences could affect treatment outcomes. Therefore, addressing this limitation in future research through subtype-stratified preclinical models and clinical trials could refine patient selection, enable personalized NSC-based therapies, and enhance therapeutic efficacy across the glioma spectrum (Baghban et al. 2020; Finch et al. 2021; Singh et al. 2025).

Emerging evidence indicates that GB stem cells exhibit distinct molecular signatures depending on their state, with proneural and mesenchymal subtypes showing differences between quiescent and proliferative phases. These findings not only provide a framework for understanding tumor heterogeneity but also suggest that recurrent GB phenotypes may result from tumor adaptation along a phenotypic continuum. The identification of two responder subtypes further underscores the role of adaptive mechanisms and differential treatment responses, offering a rationale for developing adjuvant strategies that could potentiate the effectiveness of the current standard of care. Together, these insights highlight the need to integrate stem cell biology into therapeutic design, paving the way for more precise and durable treatment approaches in this highly aggressive disease (Tanner et al. 2024).Despite these challenges, the position of NSC-based therapies in GB treatment remains highly promising. Genetic engineering strategies aimed at enhancing the tumor-homing capacity of NSCs, along with the development of biomaterials to improve their targeting and retention at tumor sites, offer significant potential to improve therapeutic efficacy. Notably, genetically modified NSCs engineered to express OVs, or anti-tumor agents have shown encouraging results in overcoming key therapeutic barriers. Additionally, a deeper understanding of the TME and the dynamic interactions between NSCs and tumor components may facilitate the development of more effective and targeted NSC-based interventions (Tang et al. 2024; Zottel et al. 2023).

Another promising avenue for future research lies in the application of personalized medicine to optimize NSC-based therapies for individual patients. Advances in genomic profiling and precision medicine now enable the tailoring of therapeutic strategies to the specific genetic and molecular landscape of each patient’s tumor. This personalized approach has the potential to enhance the efficacy of NSC therapies while minimizing off-target effects and reducing systemic toxicity (Saqib et al. 2024).

In conclusion, despite significant advancements in harnessing NSCs for GB treatment, critical challenges remain, particularly in overcoming tumor heterogeneity, improving targeting efficiency, ensuring safety, and translating preclinical success into clinical efficacy. However, ongoing developments in genetic engineering, personalized medicine, and the evolving understanding of the TME offer promising avenues for progress. Collectively, these innovations can potentially establish NSC-based therapies as a novel and effective approach for treating GB in the future.

## Data Availability

No datasets were generated or analysed during the current study.
